# Influences on advance practice nursing education to prescribe medications for opioid use disorder

**DOI:** 10.1016/j.outlook.2023.101963

**Published:** 2023-03-30

**Authors:** Bethany J. Phoenix, Matthew Tierney, Susan A. Chapman, Joanne Spetz

**Affiliations:** a Department of Community Health Systems, University of California San Francisco School of Nursing, San Francisco, CA, USA; b Department of Social and Behavioral Sciences, University of California San Francisco School of Nursing, San Francisco, CA, USA; c University of California San Francisco, Philip R. Lee Institute for Health Policy Studies, San Francisco, CA, USA

**Keywords:** Graduate nursing education, Advanced practice registered, nurses, Nurse practitioners, Addictive disorders, Opioid use disorders, Medications for opioid use, disorders, Stigma

## Abstract

**Background:**

Opioid misuse is a major public health concern in the United States. Opioid agonist medications are evidence-based treatments for opioid use disorders (OUD) that can be prescribed by advance practice registered nurses (APRNs) with prescriptive authority and appropriate training.

**Purpose:**

Article examines factors influencing preparation to provide medications for opioid use disorder (MOUD) in APRN education.

**Methods:**

Data from semi-structured interviews addressing the role of education in preparing APRNs to provide MOUD were grouped into key themes using thematic analysis. Data were collected in a mixed methods study in four states with high opioid overdose deaths whose main findings were previously published.

**Findings:**

Two overarching themes emerged: “addressing attitudes” and “curriculum change.” Sub-themes include affective barriers to providing OUD treatment; motivation to respond to the OUD crisis; and attitude change through experience with MOUD.

**Discussion and Conclusion:**

APRNs can play a key role in reducing the harms caused by OUD. Attention to attitudinal issues, such as stigma, toward people using opioids is important in educating APRNs about providing MOUD.

## Introduction

Despite the availability of evidence-based treatments to minimize harms, opioid-related morbidity and mortality have risen steadily in the United States during the 21st century (Mattson et al., 2021). Opioid-related morbidity includes infections that may occur when drugs are injected in unsterile conditions, including soft tissue infections, transmission of viral illnesses like hepatitis C and HIV, and bacterial infections. It also includes the development of opioid use disorder (OUD), with behavioral manifestations of drug craving, failure to fulfill major role obligations and repeated use in physically hazardous situations (American Psychiatric Association, 2022). In 2021, an estimated 2.5 million persons in the United States aged 12 or older met criteria for OUD (Substance Abuse and Mental Health Services Administration [SAMHSA], 2022).

The most visible source of mortality associated with opioid use is overdose deaths, which have risen steadily in the past 20 years and demonstrated a steeper increase since 2019 (Spencer et al., 2022), coinciding with the many social and economic disruptions associated with the COVID-19 pandemic. In 2020, opioid-related overdose deaths increased 30% from the prior year to 70,029, and increased by another 15% to 80,816 in 2021, accounting for 75% of all drug overdose deaths in both years (National Institute on Drug Abuse, 2022). A major driver of this increase in opioid overdose deaths has been the growing presence in the drug supply of highly potent synthetic opioids such as fentanyl, which accounted for over 82% of opioid-involved deaths in 2020 (Centers for Disease Control and Prevention, 2022). An analysis of U.S. mortality rates identified drug overdose deaths as the most rapidly increasing contributor to all-cause mortality across adult age groups, and overdose deaths were cited as being a significant contributor to the decline in U.S. life expectancy (Barbieri, 2019; Woolf & Schoomaker, 2019).

The availability of evidence-based treatment for OUDs has not been proportional to the extent of their negative health impacts on the U.S. population. Only about one-third of U.S. adults with OUD receive treatment of any kind for this condition (National Academies of Sciences, Engineering, and Medicine [NASEM], 2019), with the greatest number receiving treatment in non-medical settings from peer support groups (SAMHSA, 2022). Barriers to accessing evidence-based treatment for OUD arise from workforce issues including provider shortages; insufficient training, education and experience; lack of institutional and peer support for clinicians providing OUD services; stigma toward OUDs; and insufficient reimbursement for OUD treatment services (Bouchery & Dey, 2018).

Addiction to opioids is likely to be associated with a complex of risk and perpetuating factors including comorbid chronic pain and psychiatric disorders, family dysfunctions, restricted job and educational opportunities, involvement with the criminal justice system and access to opioids in the community (Volkow et al., 2019) that require a comprehensive treatment approach. Evidence-based behavioral treatments for substance use disorders include motivational interventions, cognitive behavioral therapies, contingency management, mindfulness-based approaches, marital and family therapies, and treatment of comorbid psychiatric conditions (Glasner & Drazdowski, 2019). Participation in self-help and peer support groups may also be an important component of a recovery plan.

Medications for opioid use disorder (MOUD), which include naloxone, naltrexone, methadone and buprenorphine, have been shown to prevent opioid-related morbidity and mortality and are recognized as a key component of treating OUD across the continuum of care (Volkow et al., 2019). Naloxone is an opioid antagonist that is increasingly used in community settings to reverse opioid overdoses and prevent related fatalities (Centers for Disease Control, 2012; LeSaint et al., 2022). Naltrexone is also an opioid antagonist with effectiveness in sustaining opioid abstinence, especially in long-acting injectable form (Krupitsky et al., 2013). Methadone, a long-acting full opioid agonist that is taken orally, has long been prescribed through specially licensed opioid treatment programs as a safer alternative to treat OUD and foster recovery. Buprenorphine, an opioid partial agonist that blocks withdrawal and reduces the risk of overdose, is also used to treat OUD. This medication can be prescribed in office-based settings by qualified practitioners, including advanced practice registered nurses (APRNs) with prescriptive authority. Because of the wider range of settings in which it can be prescribed, buprenorphine has the potential to greatly expand safe and effective OUD treatment. Methadone and buprenorphine, especially when used in long-term treatment, reduce opioid cravings and use, decrease the risk of HIV/AIDS and viral hepatitis, and reduce drug-related mortality (Ling et al, 1996; Fudala et al., 2003; Sullivan et al., 2008; Ma et al., 2019; Krawczyk et al., 2020; Santo et al., 2021).

Despite the clear need to reduce opioid-related morbidity and mortality and the established effectiveness of MOUD in doing so, MOUD are not utilized as widely as they could be. In 2021, it is estimated that only 22% of persons with an OUD received MOUD (SAMHSA, 2022). Barriers to broader use of MOUD include lack of content on evidence-based approaches to addiction treatment in health professional training programs, stigmatizing attitudes toward persons who use drugs, and regulatory barriers that limit provider capacity to prescribe MOUD (NASEM, 2019).

Fortunately, the numbers and types of health professionals who can prescribe buprenorphine are gradually increasing. Initially, only physicians were allowed to prescribe buprenorphine after completing training on this treatment modality and receiving a waiver on their DEA license. In 2016, the Congress passed the Comprehensive Addiction and Recovery Act (CARA; 2016) that allowed nurse practitioners (NPs) and physician assistants (PAs) to receive buprenorphine waivers after completing required training. The “Substance Use Disorder Prevention that Promotes Opioid Recovery and Treatment for Patients and Communities (SUPPORT) Act” extended waiver eligibility to APRNs in 2018. The increase in overdose deaths during the COVID pandemic noted above prompted the Department of Health and Human Services to issue new guidelines in 2021 exempting clinicians from training requirements for waivers to treat up to 30 patients, and the waiver requirement for clinicians prescribing buprenorphine for OUD outside licensed treatment programs was eliminated in December 2022 as part of budget reconciliation legislation.

Engagement of APRNs in providing buprenorphine treatment expands availability of this evidence-based treatment for OUD, particularly to underserved populations. There are approximately 270,000 currently active nurse practitioners (NPs) in the US (Kaiser Family Foundation, 2022), as well as other APRNs (Clinical Nurse Specialists [CNS], Certified Registered Nurse Anesthetists [CRNA], Certified Nurse Midwife [CNM]) with prescriptive authority. APRNs are more likely than physicians to practice in rural areas (Xue et al., 2019) and to serve patients without private insurance (California Health Care Foundation, 2019).

Graduate nursing education can play a key role in preparing APRNs to provide MOUD. This study examines how nurse educators and others are addressing the need to include preparation for prescribing MOUD in coursework and clinical training and the factors that support or inhibit this effort.

## Methods

### Study Overview

This report analyzes a subset of the qualitative data collected as part of a mixed methods study examining factors that supported or inhibited APRN participation in MOUD (Spetz et al., 2021). The quantitative data were analyzed to determine how state scope of practice regulation was associated with differences in MOUD waiver growth in the APRN workforce; these results have been published, reporting that physician oversight requirements have slowed growth in APRN waivers (Spetz et al., 2019, 2021). The qualitative portion of this study used a comparative case study approach to describe and compare MOUD practice by APRNs in four states, including preparation for prescribing MOUDs. The study was approved under expedited review by the university’s institutional review board.

The plan for qualitative data collection began with selecting states for comparison by identifying states with higher-than-average opioid overdose death rates and categorizing them by these factors: (a) whether NPs could prescribe buprenorphine without physician oversight; and (b) the percentage of NPs with MOUD waivers. We selected four states that varied on these two factors and represented different areas of the United States: New Mexico (autonomous APRN practice, higher percentage of waivers); West Virginia (autonomous APRN practice, lower percentage of waivers); Ohio (restricted APRN practice, higher percentage of waivers); and Michigan (restricted APRN practice, lower percentage of waivers).

Our team recruited potential interview subjects by reaching out to known contacts in each state including nursing faculty, practicing APRNs, and leaders in behavioral health agencies. After contacting these individuals, we used snowball sampling to identify additional informants in each state. Our inclusion criteria were broad since we wanted to get multiple perspectives on APRN participation in MOUD. Our subjects included APRNs who prescribed MOUD or who were interested in obtaining waivers, nursing faculty, nursing and behavioral health regulators, and agency leadership in behavioral health clinics where MOUD is offered. Our primary focus was on nurse practitioners since they were the first APRNs eligible for MOUD waivers, but we also interviewed faculty who were clinical nurse specialists as well as nurse midwives who were involved in efforts to expand the use of MOUD by childbearing people. Most of the interviews were conducted during week-long visits to the states by at least two members of the research team, with some phone interviews with informants who were not available during our visits. Altogether, we interviewed 76 key informants.

Informants were interviewed singly or in small groups by two members of the research team using semi-structured interview guides. Questions varied depending on the role and area of expertise of the person being interviewed. Researchers took detailed notes on laptop computers but did not tape-record interviews since many took place in public settings such as coffee shops or busy areas such as clinic break rooms. We provided a project description and consent form and obtained verbal consent before beginning interviews.

After each site visit, interview notes were reviewed by four members of the project team and coded for themes in the data. When all site visits were completed, the researchers met as a team to form a consensus on key themes in the data and review all notes to ensure that no additional major themes emerged. Major themes of the qualitative analysis are reported in Spetz et al. (2021).

### Analysis Focused on APRN Education

This article reports on an in-depth analysis of interviews that addressed the role of nursing education in preparing APRNs to respond to the opioid epidemic, including participation in MOUD, which emerged as a major topic area in our qualitative data. All interviews were reviewed by a member of the study team to identify those with content on the response of graduate nursing education programs to prepare students to work with patients and communities experiencing OUD. We identified 27 interviews in which the role and process of nursing education was discussed. Informants included nursing faculty, practicing APRNs, graduate nursing students and nurses leading initiatives to improve care for persons with OUD. Many of these informants had multiple roles, and the most common combination was nursing faculty who were engaged in clinical practice.

Thematic analysis (Pope & Mays, 2006) was used to group data into key themes. Major themes and subthemes are discussed, using informant quotes to represent specific perspectives.

Key elements of the team’s reflexive stance, explored in team discussions following each site visit and after data collection was completed, focused on the interpersonal and contextual domains (Olmos-Vega et al., 2022). Informants in the subset of interviews reported on here were primarily nursing faculty, similar to members of the research team conducting the interviews, thus ensuring a high degree of “shared language” and minimizing interpersonal power differentials between researchers and informants. This shared professional status may, however, have introduced some social acceptability bias to informants’ discussion of issues as attitudes toward evidence-based practices.

Contextual differences between the research team and many of our informants were related to the prevalence and social impact of opioid use in areas we visited. Since study sites were selected based on rates of opioid-related deaths in the state, informants may have experienced the impact of opioid use in their family or social networks as well as patient populations they served. This experience was not common among the research team. Our findings are also influenced by the cultural and historical context of political and health care responses to opioid use. Informants reported negotiating divergent and changing societal attitudes toward drug use in their stakeholder groups, ranging from perceptions of drug use as a personal weakness or crime to a view of drug use as a public health concern.

### Findings

Two overarching themes emerged from the data: “addressing attitudes” and “curriculum change.” A minor theme, primarily expressed by nurse midwives, was uncertainty about whether provision of MOUD care was encompassed by their APRN scope of practice.

### Addressing Attitudes

Sub-themes under the “addressing attitudes” theme include affective barriers to providing OUD treatment; motivation to respond to the OUD crisis; and the process of attitude change through exposure to clients with OUD and seeing the effects of MOUD.

### Affective Barriers

Although nursing education primarily emphasizes the knowledge and skill components of learning, nurse educator informants discussed the need to identify and address affective influences on learners’ motivation and ability to provide compassionate care to persons with substance use disorders (SUD). Affective barriers to learning about and participating in OUD treatment include stigma and negative countertransference responses. In this context, stigma is viewed as a negative societal attitude attributed to groups of people whose behavior or characteristics differ from social norms (Ahmedani, 2011). Stigma is often associated with inaccurate and judgmental beliefs about social groups. Countertransference is used in its broader sense to refer to clinicians’ emotions toward patients that result from previous life experience of the clinician and are often unconscious (Linn-Walton & Pardasani, 2014).

### Stigma

The widespread nature of stigmatizing attitudes toward drug use, and opioid use in particular, was widely cited by informants as barriers to educating students about OUD and MOUD. This includes stigma among faculty, preceptors and students that reflects the stigmatization of drug use in communities across the country. Stigma toward populations in need of MOUD was seen as due not just to their drug use, but also because of co-existing psychiatric problems that are commonly seen in these groups. A faculty member noted, “Addiction has a stigma among students...It’s a double stigma; mental illness and addiction.”

A lead faculty in a NP program noted, “I’ve had trouble getting even my own faculty waived. They are compassionate and they understand addiction as a disease, but they don’t really want to deal with the patients. There is a lot of baggage that comes with psych patients in general.” Lack of understanding of the predisposing factors for addiction was seen as contributing to negative provider attitudes. A NP informant who leads an agency providing addiction treatment services and is currently enrolled in a Psychiatric Mental Health Nurse Practitioner (PMHNP) program reports, “In my PMHNP classes, most steer clear of SUD. They don’t understand—they think, ‘These people made a choice in using drugs.’ People don’t think about how people have been living, what led them to the cycle of addiction. So much of this is about trauma. The professional community doesn’t understand this.”

Several informants noted that lack of content on substance use disorders in APRN programs in the past has contributed to stigmatizing attitudes among APRN preceptors. A clinical faculty member noted, “A lot of NPs that are already out practicing were not educated to understand SUD and SUD treatment. (One of the NPs working in an agency where NP students are placed) thinks that if you prescribe Suboxone, it will draw in people who want opioids—we will have ‘opened the gates.’”

In addition to addressing stigma toward drug-using patients, faculty members who seek to prepare APRN students to provide MOUD must also deal with the stigma that may be attached to health professionals who work with people who use drugs. In reflecting on her own previous stigmatizing beliefs, a faculty member told us, “I know my own view has changed. There also is a stigma against providers who prescribe. I remember looking down on people who worked at the methadone program.”

### Negative Countertransference

Since states in our study were chosen because of their high rates of opioid use disorder, it is not surprising that many faculty informants discussed how students’ experience with opioid use in their families or communities impact their response to education about OUD: “We work with our students on bias issues. Many come from communities with high opiate use or have a family member with addiction.” One educator cited the need to “frame students approach away from their own personal experiences with family or previous patients so they can see the patient in front of them for who they are.”

APRN students’ attitudes are shaped not only by experiences in their personal lives, but also by previous professional experiences with the harms caused by drug use. An example of this was provided by a NP educator: “If they’ve worked in maternity, they are hard to convince [to engage in MOUD training]. They’ve seen so many NAS [neonatal abstinence syndrome] babies, it triggers anger toward people who use drugs.”

The intensity of the impact of opioid use on many communities and the complex of other associated social problems evokes a feeling of being unable to make a difference in some students. “Some students get here and are just overwhelmed by it, how many people there are, how many drugs they are using.”

It should be noted that not all countertransference responses based on previous experience were negative. One faculty member noted, “The reach of the opioid epidemic has democratized students’ perception of the issue. Lots of them know someone who has died, which increases their empathy for clients and families.”

### Motivation to Respond to the Opioid Crisis

As noted above, students’ personal and professional experiences often motivated them to pursue additional knowledge and training in OUD treatment. Most of our informants reported that their students were willing, and sometimes enthusiastic, to learn more about MOUD and potentially become MOUD providers since “they’ve been in practice, and they see the need.” Faculty whose curriculum included MOUD waiver training often reported that a substantial portion of their classes planned to provide MOUD after graduation. However, educators reported difficulty finding clinical placements that included MOUD provision. One faculty member noted, “Students have an open attitude about it [MOUD] and want to learn more, especially as they get more exposure. Their frustration is that they are not seeing MOUD modeled in practice anywhere.”

### Attitude Change Through Exposure to MOUD

Across informant groups in our larger study, a prominent theme was how people’s attitudes toward MOUD changed when they had exposure to this treatment modality and could see how patients’ lives changed when they received MOUD. Many practitioners and nursing faculty acknowledged that they had originally been biased toward abstinence-based treatment, viewing medication treatments such as methadone and buprenorphine as “substituting one addiction for another.” A faculty in a NP program whose clinical practice was in a federally qualified health center reported, “A lot of my patients were struggling with addiction. . . When CARA passed, I wanted to be part of the solution, although I’ll admit I was not convinced MOUD was a great long-term solution. I took the training, and that opened my eyes to the potential positive impact.” This educator subsequently became a champion for including MOUD waiver training in her university’s NP programs.

Faculty also reported changes in student attitudes through their clinical exposure to drug-using clients. “I think a lot of it is the way students perceive people with addictions. The first thing they think about somebody addicted to heroin is a person who is strung out and on the streets, and usually that’s not true. We get older people who were on prescription meds and when that prescription ended, they started smoking heroin [to manage the pain.] I have a lot [of patients] who come from the judicial system, but a lot do not. You don’t know who might be a lawyer or a business owner. It’s hard to teach that in the curriculum as far as didactic. It comes in the practice when they get the hands on and make the connection with the person—the bias has gone away. They hear ‘I didn’t choose to become an addict.’ Then students have the ability to personalize that and their attitude changes.”

Another faculty member reports, “I have seen a change among students in terms of their acceptance of MOUD…. The NP student shadowing me is a PICU nurse who has had a lot of hard feelings about SUDs because of what she has seen with her pediatric patients. But she is seeing this differently now…. I think the impact is the affective change from hearing the patients’ stories. The cognitive change of receiving information also helps.”

### Scope of Practice Concerns

Many of our informants commented on the challenges of providing care to patients with complex addictive and mental health disorders that are compounded by structural vulnerabilities such as poverty, discrimination and exposure to violence. Although some faculty and clinicians expressed concerns about whether primary care NP programs included sufficient content on mental health and substance use disorders to prepare their graduates to address the needs of such complex populations, the only concerns we heard from these informants that directly related to scope of practice involved concerns about regulatory scrutiny of controlled substance prescriptions. Since NPs often practice in sites serving vulnerable and complex patient populations, managing complex health problems has become accepted as part of their scope of practice.

Certified Nurse Midwife (CNM) informants who are leading initiatives to expand care for pregnant people who use opioids reported that, in some rural communities, CNMs are the only health care providers who are willing to prescribe MAT. However, they also described some concerns expressed by their CNM colleagues about whether providing MOUD to pregnant patients is within their scope of practice: “For midwifery, particularly for nurse-midwifery, there is the belief that ‘we take care of normal pregnancies’ and when it gets complicated you bring in a doc.” Another CNM noted, “It has been a challenge for midwifery to define what ‘low-risk care,’ which is supposed to be focus of midwifery, means. It’s clear what is medical high risk, but some CNMs are reluctant to take on patients who have complex health and psychosocial problems, thinking ‘maybe it’s not within my scope.’”

### Curriculum Change

Another major theme in our data involved efforts at curriculum change to better prepare students to care for drug-using patients and their families. The impetus for curriculum enhancement often came from a nurse faculty champion who mobilized colleagues to participate. Much of the curriculum work was supported by federal funding to these informants’ institutions, often in the form of interprofessional training grants to enhance education on addictive disorders. In some cases, SUD-focused courses included faculty and students from multiple disciplines.

Although APRN programs integrated didactic addictions content in a variety of different courses, it was most commonly emphasized in pharmacology course-work. Several informants also described how SUD content was incorporated into assessment and management courses. About half the programs we met with either required MOUD waiver training or were planning to implement such a requirement soon. These programs incorporated available online MOUD waiver trainings available through government agencies or professional organizations. One nursing school was planning to reintroduce a minor focused on substance use and another offered a post-graduate PMHNP certificate program that was advertised as having a focus on addiction.

Arranging clinical training opportunities where students could learn skills in addiction assessment and management was often more challenging than adding didactic content. Although several programs reported requirements for students to spend from 20 to 100 hrs of clinical training in addiction settings or with providers who practice MOUD, others noted difficulty finding this kind of experience for their students: “They’re mostly seeing referrals to specialized addiction treatment.”

In addition to fostering knowledge and skill development, nurse educators incorporated pedagogical strategies to address the affective component of learning. Clinical simulations were the most commonly mentioned strategy to increase affective engagement. In addition to low-fidelity simulations using an online simulation program, one NP program reports, “We work with a peer recovery group who come in as actors. They role-play drug-seeking. They tell their own stories. The students say this was the best learning experience they’ve ever had.”

Programs also included assessments to measure stigma before and after MOUD waiver training and assess students’ intent to provide MOUD after graduation. One NP program requires students engaged in MOUD waiver training to do journaling on addiction-related topics, including why they would or would not consider being a buprenorphine provider.

### Discussion and Recommendations

Given the increases in OUD-related morbidity and mortality and the multiple negative effects of addictions on population health in the United States, it is important for all nurses to receive education on the assessment and treatment of SUDs, including MOUD. This study adds to the growing body of literature on how SUD content is incorporated in graduate nursing programs (Campbell-Heider et al., 2009; Finnell et al., 2018; Smothers et al., 2018), and specifically how programs integrate curricular content to prepare APRNs for providing MOUD (Barcelona et al., 2022; Compton & Blacher, 2020; Kameg et al., 2018). With some exceptions, curricular attention devoted to topics related to addictions has been minimal, considering the public health impact of addictive disorders (Aronowitz et al., 2021; Smothers et al., 2018). Compton and Blacher (2020) regard nursing curricula as being “shockingly sparse” in content necessary to prepare nurses with the competencies needed to care for persons experiencing, or at risk for, OUDs. However, when SUD content is included in nursing curricula, there is substantial evidence that it positively affects students’ knowledge, attitudes and skills (Smothers et al., 2018).

The urgency of threats to public health posed by the opioid crisis in their states and federal funding to expand education on OUDs for health care providers were motivating factors for the nurse educators we interviewed to develop or enhance curriculum on addictive disorders. Many collaborated with colleagues from other disciplines at their universities, including pharmacy and medicine. Faculty informants leading curricular change often described the need to educate themselves about addictions and address their own stigmatizing attitudes toward people who use drugs, which highlights the need for faculty development that emphasizes self-reflection and attitudinal change in addition to increasing knowledge about OUDs. Our findings are consistent with other studies demonstrating increased confidence in the efficacy of MOUDs in providers who have experience with this treatment modality (Andraka-Christou et al., 2022).

The incorporation of existing online MOUD waiver trainings into graduate nursing curricula provides a model for how additional curricular materials developed for health professionals’ addiction education might be used. The “Arizona Pain and Addiction Curriculum” is an example of a curricular template developed by an interdisciplinary group of health care providers that can be adapted for use by diverse educational programs (Villaroel et al., 2020). Core competencies for substance use assessment and management provide guidance for competency development specific to nursing (Finnell et al., 2019), and these competencies are perhaps even more important now that clinicians are not required to have a waiver to offer MOUD to their patients.

The multiple narratives we heard about attitudinal change and greater understanding and empathy for people who use drugs emphasize the need for attention to the affective component of addiction education. Adequate opportunities to process direct clinical experience, dialogues with peer recovery specialists, clinical simulations, and reflective exercises such as journaling can be effective pedagogical strategies to combat stigma. Support for students’ self-care and opportunities to express strong emotions such as grief, frustration and hopelessness are also important. With the increase in opioid-related mortality in recent years and the complex mental health and psychosocial challenges seen in people who misuse opioids, APRN students may feel overwhelmed or ineffectual when they do not see positive results. They are also at risk for compassion fatigue arising from their empathic engagement with patients struggling with addictions and multiple life stressors.

## Limitations

This analysis includes a cross-section of nursing faculty in several geographic regions of the United States who are engaged in efforts to increase addictions content in their APRN programs but cannot be considered representative of such efforts in nursing programs throughout the country. Since our study chose four states where opioid overdose death rates were high, it is likely that the sense of urgency about addressing the opioid epidemic was higher in these states than in areas of the United States where opioid-related deaths are less frequent. In addition, our sampling strategy focused on finding informants who were knowledgeable about the role of nursing in responding to the opioid epidemic so many of our subjects were leaders in mobilizing the nursing response.

Since data for this analysis were obtained as part of a comparative case study on how multiple factors affect APRN participation in MOUD, attention to APRN education was less systematic than if this had been the sole or primary focus of our research. In many interviews with faculty, we discussed their roles as both practitioners and educators, which limited the time we were able to spend on questions related to curriculum, clinical training, and attitudinal change.

Due to the broad-based scope of our inquiry and semi-structured nature of the interviews, we were unable to use data saturation as an endpoint for our exploration of APRN education to prescribe MOUDs. Data for this analysis were selected from among completed interviews based on their relevance to this topic. Thus, our findings should be considered preliminary. Further research focused specifically on educational preparation for APRNs to prescribe MOUDs, particularly as the regulatory landscape for OUD treatment changes, is needed to confirm our findings.

## Conclusion

Nurses can play a key role in addressing the multiplicity of health, psychosocial and economic problems caused by the U.S. opioid epidemic by being prepared to intervene at every stage of prevention and treatment. Providing MOUD is an evidence-based treatment that APRNs with appropriate educational preparation and prescriptive authority can provide to minimize opioid-related harms. This report discusses barriers and motivating factors for implementing OUD education in advanced practice nursing programs. These including the need for attention to attitudinal issues such as stigma and hopelessness, as well as capitalizing on students’ motivation to address the needs of persons with OUDs. Informants also identified the need for greater attention to the complex psychiatric and psychosocial issues associated with OUDs in APRN curricula.
